# The changing characteristics of patients with chronic hepatitis C prescribed direct acting antiviral medicines in general practice since listing of the medicines on the Australian Pharmaceutical Benefits Scheme

**DOI:** 10.1002/jgh3.12593

**Published:** 2021-06-18

**Authors:** Doreen Busingye, Kendal Chidwick, Vanessa Simpson, Jonathan Dartnell, Gregory J Dore, Anne Balcomb, Suzanne Blogg

**Affiliations:** ^1^ NPS MedicineWise Sydney New South Wales Australia; ^2^ The Kirby Institute University of New South Wales Sydney Sydney New South Wales Australia; ^3^ Prince Street Medical Practice Orange New South Wales Australia

**Keywords:** direct acting antiviral, general practice, hepatitis C, patient characteristics, prescribing

## Abstract

**Background and Aim:**

The primary objective of this study was to determine whether the characteristics of patients prescribed direct acting antiviral (DAA) medicines have changed since initial listing of the medicines on the Australian Pharmaceutical Benefits Scheme (PBS).

**Methods:**

A cross‐sectional study was conducted using data from MedicineInsight, an Australian database of general practice electronic health records, from March 2016 to August 2018. We compared sociodemographic, comorbidity, and clinical characteristics of patients aged at least 18 years who were prescribed at least one DAA in the first 4 months of PBS listing in 2016 with those prescribed at least one DAA in 2018.

**Results:**

There were 2251 eligible adult patients prescribed a DAA during the study period, 62% were men and 59% were aged 50 years and older. Patients prescribed DAA medicines initially were older (aged ≥50 years: 67.9% *vs* 49.3%; *P* < 0.001), and more likely to have liver cirrhosis (14.2% *vs* 8.4%; *P* = 0.01) and an aminotransferase to platelet ratio index (APRI) score >1 (20.4% *vs* 8.9%; *P* < 0.001) than those prescribed DAA medicines in 2018. A greater proportion of patients in regional/remote (46.5% *vs* 35.6%; *P* < 0.001) and socioeconomically disadvantaged areas (44.4% *vs* 34.5%; *P* = 0.003) accessed treatment in 2018 compared with 2016.

**Conclusions:**

Despite evidence of decreasing uptake of DAA medicines across Australia, this study indicates broadened uptake among younger age groups and those residing in regional/remote and socioeconomically disadvantaged areas since 2016. While uptake of DAA medicines in some population subgroups appears to have improved, continuous efforts to improve uptake across the Australian population are essential.

## Graphical abstract

Despite evidence of decreasing uptake of direct acting antiviral (DAA) medicines across Australia, there has been broadened uptake among younger age groups and those residing in regional/remote and socioeconomically disadvantaged areas since 2016. Although uptake of DAA medicines in some population subgroups appears to have improved, continuous efforts to improve uptake across the Australian population are essential.

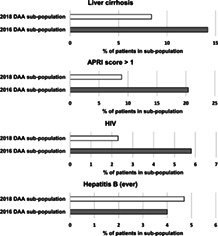

## Introduction

Chronic hepatitis C (CHC) is a major public health threat, with 71 million people estimated to have the condition globally.^1^ In Australia, over 182 000 people were estimated to have had CHC at the end of 2017.^2^ People with CHC are at risk of progressive liver fibrosis leading to cirrhosis, liver failure, and hepatocellular carcinoma (HCC). Until direct acting antiviral (DAA) medicines were listed on the Pharmaceutical Benefits Scheme (PBS) on first March 2016, only 20% of people living with CHC infection had ever received treatment, which had limited effectiveness and poor tolerability.^3^ New orally administered DAA treatments have cure rates of over 95%, are largely well tolerated, and are available at low cost to Medicare‐eligible Australians.^3^, ^4^ The majority of people with CHC in Australia can potentially be cured, reducing the risk of liver disease and other serious complications.

Australia maintains universal, subsidized healthcare for all eligible citizens and permanent residents. General practitioner (GP) consultations are subsidized through the Medicare Benefits Schedule (MBS)^5^ and some prescription medicines are subsidized through the PBS.^6^ Private prescriptions can be written for medicines that are not listed on the PBS but approved for use in Australia and the patient pays the entire cost out‐of‐pocket.^6^, ^7^

Since the listing of the new DAA medicines on the PBS, there was an initial substantial uptake in DAA treatment in the first 4 months followed by a steadily decreasing trend of dispensing.^8^ The initial increase in DAA uptake likely reflects a “warehouse” effect, where many patients awaiting access to DAA medicines were treated in the initial months of PBS listing.^9^ Treatment with DAA medicines was likely prioritized to patients with advanced fibrosis, who are at a greater risk of developing liver‐related complications.^10^ More accessible patients identified as having CHC were also most likely to have been treated.^8^

While DAA treatment uptake rates can be adequately described using PBS data,^8^ general practice data allow the characterization of the comorbidities and clinical characteristics of patients prescribed DAA therapy in general practice. The primary aim of this study was to use data from MedicineInsight, an Australian general practice database, to determine whether the sociodemographic, comorbidity, and clinical characteristics of patients prescribed DAA medicines by GPs have changed over time since the initial PBS listing of the medicines and to assess re‐treatment rates for DAA regimens.

## Methods

### Design and data source

A cross‐sectional study was conducted using MedicineInsight data from 1 March 2016 to 31 August 2018, collected from 359 general practice sites that met the data quality requirements (described elsewhere).^11^

MedicineInsight is a national general practice data program developed and managed by NPS MedicineWise with funding support from the Australian Government Department of Health.^11^ MedicineInsight extracts and collates longitudinal, de‐identified patient health records, including demographics, encounters (excluding progress notes), diagnoses, prescriptions, and pathology tests from the clinical information systems, Medical Director and Best Practice. MedicineInsight includes records for over 3.5 million regular patients (approximately 15% of the Australian population) from more than 5000 GPs in over 700 general practices across Australia (as at 1 July 2019). When compared with MBS data, the characteristics of regularly attending MedicineInsight patients are broadly comparable to those patients who visited a GP in 2016/2017.^11^

### Participants

Patients were included if, at the time of data extract, they were aged at least 18 years, had valid information for age and sex, had at least one clinical encounter during the study period at an included general practice site, and had at least one prescription for a DAA medicine recorded during the study period. Subpopulations drawn from the same 359 general practice sites included:

1. The 2016 DAA subpopulation—patients who had at least one prescription for a DAA recorded during the first 4 months after PBS listing (1 March 2016 to 30 June 2016)
2. The 2018 DAA subpopulation—patients who had at least one prescription for a DAA recorded during 2018 to the end of the study (1 January 2018 to 31 August 2018). A longer time period was chosen in 2018 compared with 2016 to increase the sample size.
3. The re‐treatment subpopulation—patients who had at least one prescription for a DAA recorded during 1 March 2016 to 31 December 2017, allowing at least 8 months from the initial script (until 31 August 2018) for re‐treatment to be recorded if it occurred.

### DAA medicines

MedicineInsight prescribing information includes whether the prescription is marked as eligible for subsidy under the PBS or the Repatriation Pharmaceutical Benefits Scheme (RPBS), which is available to specified war veterans and their families, or not eligible (private). Medicines were identified using the Anatomical Therapeutic Chemical (ATC) Classification System code, “medicine active ingredient,” “medicine name,” and, where required, the route of administration and strength. Patients were defined as having had a prescription for DAA if they had at least one record of an issued prescription containing a DAA during the study period. Patients with a DAA re‐treatment are those who had other DAA regimens recorded (the same or different) after the initial script during the study period. The full search list of DAA medicines is provided in Table S1, Supporting information.

### Variables

Sociodemographic characteristics included age (based on year of birth), sex, state/territory, socio‐economic indexes for areas (SEIFA) and remoteness. State/Territory, remoteness, and SEIFA were based on the patients' residential postcodes. Remoteness was determined in accordance with the Australian Bureau of Statistics (ABS) geographical framework “Remoteness Areas.”^12^ SEIFA was determined according to the ABS Index of Relative Socio‐Economic Advantage and Disadvantage (IRSAD).^13^ IRSAD is an indicator of relative economic and social advantage/disadvantage position within an area compared with the rest of the country.

Additional variables included CHC complications including liver cirrhosis and hepatocellular carcinoma (HCC), and comorbid conditions such as Hepatitis B and HIV. Patients were defined as having any of these conditions if they had a relevant coded (Docle, Pyefinch) or free text entry in one of the three diagnosis fields—diagnosis, reason for encounter, or reason for prescription—ever recorded at any time from the patient's earliest record up to the download date. The clinical definitions for the relevant conditions are shown in Table S2. We calculated aminotransferase (AST) to platelet ratio index (APRI) scores for patients in the 2016 subpopulation and the 2018 subpopulation. APRI scores were calculated according to the formula,^14^ “APRI = [(AST level (IU/L) ÷ AST (upper limit of normal, i.e. 40 IU/L)) ÷ platelet count (10^9^/L)] × 100,” using the most recent AST and platelet test results available in the 2 years prior to 30 June 2016 for the 2016 subpopulation and the 2 years prior to 31 August 2018 for the 2018 subpopulation.

### Statistical analysis

Descriptive statistics were used to describe the distribution of sociodemographic and comorbidity/clinical characteristics including frequencies, percentages and associated 95% confidence intervals (CIs) (using robust errors to adjust for clustering by practice), means, and medians. A two‐sided *P* < 0.05 from a two‐sample test of proportions was used to determine statistically significant differences between time periods. To preserve the privacy of individuals, results reported for 1–4 patients are reported as <5. Data management and analyses were conducted using SAS version 9.4 (SAS Institute Inc., Cary, NC, USA).

### Ethics

Approval to conduct this study was granted by the Royal Australian College of General Practitioners National Research and Evaluation Ethics Committee (NREEC 19–004, protocol SBO3102) and the MedicineInsight Independent Data Governance Committee (reference number: 2018–035).

## Results

### Patient profiles

There were 2251 eligible adult patients who had at least one recorded prescription for a DAA during the study period; 62% were men, 59% were 50 years and older, and 55% were from major cities (Table 1). The most commonly prescribed regimen was sofosbuvir with ledipasvir (44%), followed by sofosbuvir (31%) and daclatasvir (27%) and sofosbuvir with velpatasvir (20%) (Table S3).

**Table 1:** Sociodemographic characteristics of patients with a DAA prescription recorded during the study period

	DAA study population (*N* = 2251)		2016 DAA subpopulation(*N* = 452)		2018 DAA subpopulation(*N* = 428)	
Characteristic	*N*	% (95% CI)	*n*	% (95% CI)	*n*	% (95% CI)
Age group (years)						
18–29	87	3.9 (2.9–4.8)	7	1.5 (0.4–2.7)	22	5.1 (2.8–7.5)
30–39	301	13.4 (11.5–15.2)	44	9.7 (6.9–12.6)	79	18.5 (13.9–23.0)
40–49	533	23.7 (21.8–25.6)	94	20.8 (16.5–25.1)	116	27.1 (22.6–31.6)
50–59	756	33.6 (31.6–35.6)	186	41.2 (36.6–45.7)	125	29.2 (24.5–33.9)
60–69	494	21.9 (19.7–24.2)	107	23.7 (19.5–27.9)	71	16.6 (11.9–21.3)
70–79	61	2.7(2.0–3.4)	10	2.2 (0.7–3.7)	7	1.4 (0.3–2.5)
80–89	15	0.6 (0.3–1.0)	<5	na	6	1.6 (0.4–2.9)
90+	<5	na	<5	na	<5	na
Sex						
Female	852	37.8 (35.0–40.7)	164	36.3 (31.1–41.4)	165	38.6 (34.1–43.1)
Male	1399	62.2 (59.3–65.0)	288	63.7 (58.5–68.9)	263	61.4 (57.0–65.9)
Remoteness						
Major city	1248	55.4 (45.3–65.6)	291	64.4 (51.7–77.0)	229	53.5 (42.9–64.1)
Inner regional	716	31.8 (22.6–41.0)	114	25.2 (14.1–36.4)	148	34.6 (24.7–44.4)
Outer regional	263	11.7 (6.8–16.6)	42	9.3 (3.2–15.4)	48	11.2 (6.0–16.4)
Remote/very remote	24	1.0 (0.0–1.3)	5	1.1 (0.8–1.7)	<5	na
State/territory						
Australian Capital Territory	19	0.8 (0.0–1.6)	<5	na	9	2.1 (0.2–4.0)
New South Wales	1079	47.9 (37.3–58.6)	223	49.3 (34.6–64.1)	182	42.5 (31.9–53.1)
Northern Territory	17	0.8 (0.0–1.5)	<5	na	<5	na
Queensland	321	14.3 (8.8–19.7)	65	14.4 (6.9–21.9)	80	18.7 (11.0–26.4)
South Australia	11	0.5 (0.0–0.9)	<5	na	<5	na
Tasmania	132	5.9 (2.8–8.9)	11	2.4 (0.6–4.2)	21	4.9 (2.0–7.8)
Victoria	361	16.0 (9.3–22.8)	98	21.7 (11.0–32.3)	76	17.8 (10.3–25.2)
Western Australia	311	13.8 (5.5–22.1)	43	9.5 (3.7–15.3)	56	13.1 (2.9–23.3)
SES (SEIFA quintiles)						
1 (least advantaged)	437	19.4 (12.9–25.9)	65	14.4 (7.9–20.9)	91	21.3 (13.8–28.7)
2	530	23.5 (13.5–33.6)	91	20.1 (9.3–30.9)	99	23.1 (11.7–34.5)
3	523	23.2 (15.4–31.1)	83	18.4 (9.1–27.6)	109	25.5 (17.0–33.9)
4	529	23.5 (12.6–34.4)	135	29.9 (12.9–46.8)	90	21.0 (12.2–29.9)
5 (most advantaged)	228	10.1 (5.9–14.4)	76	16.8 (8.4–25.2)	39	9.1 (4.3–13.9)
Missing	<5	na	<5	na	‐	

CI, confidence interval; DAA, direct acting antiviral; na, not applicable; SEIFA, socio‐economic indexes for areas; SES, socioeconomic status.

A slightly lower number of patients were prescribed a DAA over the 8‐month period in 2018 (*n* = 428) than during the first 4 months after PBS listing in 2016 (*n* = 452); equating to just over a 50% reduction in the monthly prescribing rate. Across both time periods, a greater proportion (>60%) of those prescribed DAA medicines were men (Table 1). Patients who were prescribed DAA medicines in 2016 were older than those prescribed DAA medicines in 2018 (mean age: 53.4 years *vs* 49.2 years; median age: 55.0 *vs* 49.0 years, respectively). A greater proportion of patients prescribed DAA medicines in 2018 were aged <50 years than those prescribed in 2016 (50.7% *vs* 32.1%; *P* < 0.001) (Table 2). Additionally, a greater proportion of patients in regional and remote areas (46.5% *vs* 35.6%; *P* < .001) and those from socioeconomically disadvantaged areas (44.4% *vs* 34.5%; *P* = 0.003) accessed treatment in 2018 compared with 2016 (Table 2).

**Table 2:** Characteristics of patients in the 2016 DAA subpopulation and 2018 DAA subpopulation

	2016 DAA population(*N* = 452)	2018 DAA population(*N* = 428)	
Characteristic	Number (%)^†^	Number (%)^†^	*P* value^‡^
Age group (years)			
<50	145 (32.1)	217 (50.7)	<0.001
≥50	307 (67.9)	211 (49.3)	<0.001
Remoteness			
Major city	291 (64.4)	229 (53.5)	0.001
Regional/remote	161 (35.6)	199 (46.5)	<0.001
SES (SEIFA quintiles)			
Low (1 and 2)	156 (34.5)	190 (44.4)	0.003
Middle (3)	83 (18.4)	109 (25.5)	0.01
High (4 and 5)	211 (46.7)	129 (30.1)	<0.001
CHC complication			
Liver cirrhosis	64 (14.2)	36 (8.4)	0.01
Hepatocellular carcinoma	14 (3.1)	<5 (na)	
APRI score^§^			
APRI ≤1	190 (42.0)	170 (39.7)	0.49
APRI >1	92 (20.4)	38 (8.9)	<0.001
Comorbid condition			
HIV	26 (5.8)	10 (2.3)	0.01
Hepatitis B (ever)	18 (4.0)	20 (4.7)	0.61

^†^ Data are presented as *n* (%). Proportions were calculated including those with missing or not assessable data.

^‡^ Two‐sided *P* value from a two‐sample test of proportions comparing the two DAA populations.

^§^ Data for 170 patients in the 2016 DAA population and 220 patients in the 2018 DAA population were not assessable for APRI score.

APRI, aminotransferase to platelet ratio index; CHC, chronic hepatitis C; DAA, direct acting antiviral; HIV, human immune deficiency virus; na, not applicable; SEIFA, socio‐economic indexes for areas; SES, socioeconomic status.

**Figure 1:**
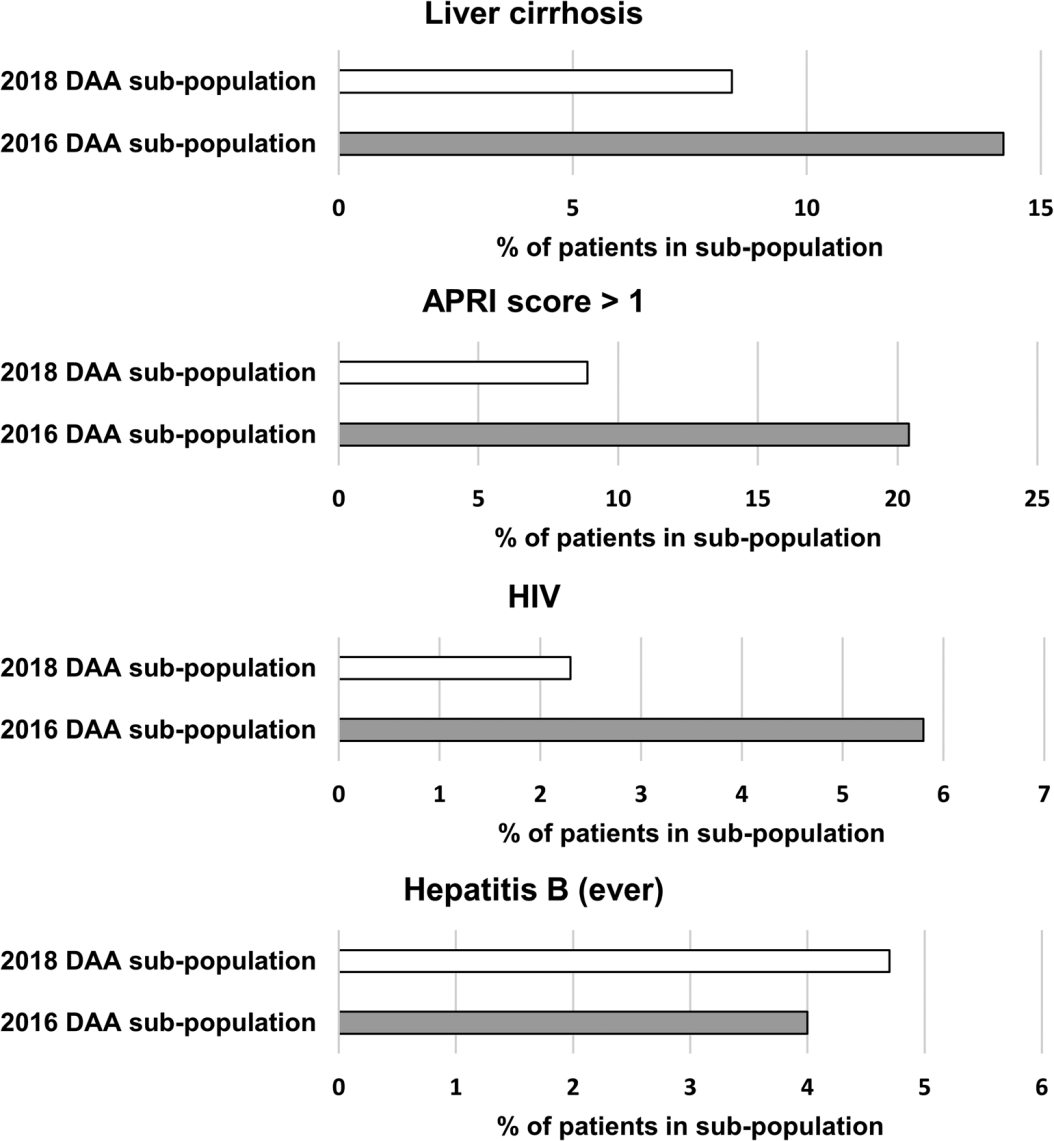
Comorbidity and clinical characteristics of patients in the 2016 DAA subpopulation (*n* = 452) and 2018 DAA subpopulation (*n* = 428). APRI, aminotransferase to platelet ratio Index; CI, confidence interval; DAA, direct acting antiviral; HIV, human immune deficiency virus.

Patients prescribed a DAA in 2016 were more likely to have a recorded diagnosis of liver cirrhosis than those in 2018 (14.2% *vs* 8.4%; *P* = 0.01) and were more likely to have an APRI score >1 (20.4% *vs* 8.9%; *P* < 0.001), indicating high risk of cirrhosis (Table 2 and Fig. 1). The proportion of patients with HIV prescribed DAA medicines was greater in 2016 than in 2018 (5.8% vs. 2.3%; *P* = 0.01) (Table 2 and Fig. 1).

### Re‐treatment (multiple DAA regimens)

Of 1848 patients who received at least one DAA prescription between 1 March 2016 and 31 December 2017, 130 patients (7%) had another course of DAA recorded at the same practice after their first DAA treatment. Most patients (99 of 130; 76%) had the same subsequent DAA regimen recorded as the first (Table 3).

**Table 3:** Number and percentage of chronic hepatitis C patients in the retreatment subpopulation who were re‐treated with a DAA, 1 March 2016 to 31 August 2018

First DAA regimen recorded during the study period	Number of patients prescribed the DAA	Patients retreated, *n* (%)(*N* = 1848)	Patients retreated with a different regimen, *n* (%)(*N* = 130)
Ledipasvir with sofosbuvir	902	52 (5.8)	8 (15.4)
Sofosbuvir and daclatasvir	561	32 (5.7)	7 (21.9)
Sofosbuvir with velpatasvir	162	12 (7.4)	<5 (na)
Grazoprevir with elbasvir	98	11 (11.2)	<5 (na)
Sofosbuvir	77	16 (20.8)	8 (50.0)
Daclatasvir	19	5 (26.3)	<5 (na)
Paritaprevir with ritonavir, ombitasvir, and dasabuvir	14	0 (0.0)	0 (0.0)
Paritaprevir with ritonavir, ombitasvir, dasabuvir, ribavirin	9	<5 (na)	0 (0.0)
Glecaprevir with pibrentasvir	6	<5 (na)	0 (0.0)
Total	1848	130 (7.0)	31 (23.8)

DAA: direct acting antiviral; na: not applicable.

## Discussion

Our findings demonstrate differences in characteristics among patients prescribed DAA medicines in the first 4 months following the March 2016 PBS listing compared with those treated in 2018. There was evidence of a shift in prescribing, with patients prescribed DAA medicines in 2018 being significantly younger than those in 2016. The proportion of individuals aged less than 50 years who were prescribed DAA medicines increased significantly from 32% in 2016 to 51% in 2018. These findings may reflect the “warehouse” effect where patients who were already diagnosed with CHC awaited access to DAA treatment.^9^

Consistent with findings from PBS data,^10^ treatment rates were initially higher for patients with an APRI score >1, suggesting advanced liver disease, including liver cirrhosis and high risk of cirrhosis. Given DAA medicines are very effective and well tolerated compared with previous therapies,^3^, ^4^ it is reasonable that treatment uptake would initially be higher for patients with advanced disease to mitigate further complications and hepatitis C virus (HCV)‐related mortality. Investigators from the United States showed that age, having cirrhosis, and a history of liver transplant were positively associated with initial uptake in the first 2 years of DAA availability.^15^

Our results indicate that initial access of DAA medicines was mainly in urban settings. However, more patients in regional or remote and socioeconomically disadvantaged areas accessed treatment in 2018 compared with 2016. This finding indicates broadened and potentially more equitable access in Australia over time. Expanding the role of management of CHC and prescribing eligibility to GPs may be responsible for improved access to DAA treatments in populations with limited access to specialist care. It has been shown that DAA regimens were mostly prescribed by specialists during the initial year of listing and by 2018 most prescribing was done by GPs.^8^, ^10^ Despite these encouraging findings of expanded access among some groups, overall uptake of DAA treatment across Australia declined steadily after 2016 and remained stable during 2017 and 2018.^8^

Authors of a recent study suggested that if Australia is to achieve the World Health Organization's elimination targets, increased identification and testing of people exposed to HCV is required^16^ as well as improved access to DAA treatment. Continued support for testing and managing HCV in primary care is required. Implementing procedures such as recall systems or other support (e.g. practice nurses) to assist with screening and following up patients for diagnosis, treatment, or review is vital. Following a number of Australian educational initiatives, awareness among GPs is growing, although some may not be experienced or prepared to take on an enhanced role (especially where they perceive few patients in their practice require HCV management). Continued support from specialists, such as the REACH‐C project, and training programs provided by the Australasian Society for HIV, Viral Hepatitis and Sexual Health Medicine (ASHM) are ideal to increase the GPs knowledge and experience enabling them to prescribe treatment for HCV.^17^, ^18^ In a survey of GPs from Victoria, Wade and colleagues found that most GPs reported an interest in prescribing DAA medicines for HCV and were willing to undertake educational activities to further their knowledge. In the same study, GPs with high HCV caseloads had more knowledge of HCV and were more likely to prescribe DAA medicines.^19^

While barriers to increased uptake in primary care remain, another strategy that could be considered is providing incentives for both the practitioners and patients. Some local health districts in New Zealand have implemented incentives for GPs who provide HCV treatment in a primary care setting,^20^ and in Australia this strategy is being used on an ad hoc basis for some patient groups. Models of care using nurse practitioners upskilled in the treatment of HCV and telehealth services supported by specialists may help improve treatment rates, particularly in remote areas with limited access to GP or specialist services.^21^, ^22^ Findings of a recent study from South Australia showed that models of care that include GPs or mixed consultant nurse models are cost‐effective ways of promoting HCV treatment uptake.^23^ Continuous monitoring of treatment uptake and outcomes in primary care setting using programs such as the REACH‐C project and MedicineInsight are ideal for providing crucial data to inform and help sustain these efforts.

The re‐treatment rate in this study was 7%, but the majority (76%) of these patients had the same DAA regimen recorded as the initial regimen. Although MedicineInsight data provide no information for the reasons of re‐treatment, this would suggest re‐treatment for either early treatment discontinuation or reinfection, rather than posttreatment relapse. Access to re‐treatment, particularly for reinfection, is a key element of HCV elimination strategies, although this has an impact on the cost‐effectiveness of the drugs as initially assessed by the Pharmaceutical Benefits Advisory Committee.

The strengths of the MedicineInsight data include the large size and national coverage of general practices, and the regular patients are broadly representative of the Australian patient population.^11^ Unlike other national prescribing datasets in Australia, these data contain diagnoses and other clinical information recorded in general practice. These data have limitations, in addition to those inherent in routinely collected data described elsewhere.^11^ Patients may have been prescribed DAA medicines by specialists or GPs at other practices not captured in this study. Thus, these results may be an underestimate of the proportion of patients who had re‐treatment. Given that the data do not incorporate specialist treatment, it is likely that temporal trends to a younger age and earlier disease seen in the MedicineInsight DAA population would be even more pronounced in the total DAA treated population, as specialists care for patients with advanced liver disease. We assume most patients in the 2016 DAA cohort were new to DAA therapy, however, it is possible that some patients in the 2018 cohort may have been on previous DAA therapy. MedicineInsight contains GP prescribing information and it is not known if the medicines are dispensed or used. For privacy reasons, MedicineInsight does not include data from progress notes, which may contain further clinical information. Because practices that participate in MedicineInsight are not randomly selected, there is potential for selection bias; although not formally assessed, we might assume that MedicineInsight practices differ systematically from non‐MedicineInsight practices in terms of being larger, more likely to use electronic health records, and more likely to participate in quality improvement programs.

## Conclusions

We have demonstrated that patients who were prescribed DAA treatment in the initial months of listing were more likely to be older, have advanced disease, and better access to treatment. Although these data suggest a broadening of DAA treatment over time among younger age groups, and patients residing in regional and socioeconomically disadvantaged areas, continuous efforts to improve identification of HCV and treatment uptake across the Australian population are essential.

## Supporting information


**Table S1**. Direct acting antiviral (DAA) medication list.
**Table S2**. Clinical definitions used to identify MedicineInsight patients.
**Table S3**. Patients prescribed individual DAA medicines during the study period (1 March 2016–31 August 2018).
