# Supplementary material for: The changing characteristics of patients with chronic hepatitis C prescribed direct acting antiviral medicines in general practice since listing of the medicines on the Australian Pharmaceutical Benefits Scheme
Source: JGH Open. 2021 Jun 18;5(7):813–9. doi: 10.1002/jgh3.12593 (PMC8264235; doi:10.1002/jgh3.12593)
Supplement: Supplementary file 1 — Table S1. Direct acting antiviral (DAA) medication list. Table S2. Clinical definitions used to identify MedicineInsight patients. Table S3. Patients prescribed individual DAA medicines during the study period (1 March 2016–31 August 2018). [file JGH3-5-813-s001.docx]

**Supporting information**

**The changing characteristics of patients with chronic hepatitis C prescribed direct acting antiviral medicines in general practice since listing of the medicines on the Australian Pharmaceutical Benefits Scheme**

Doreen Busingye,* Kendal Chidwick,* Vanessa Simpson,* Jonathan Dartnell,* Gregory J. Dore,^†^ Anne Balcomb,^‡^ Suzanne Blogg*

* NPS MedicineWise, Sydney, New South Wales, Australia

^†^ The Kirby Institute, University of New South Wales Sydney, Sydney, New South Wales, Australia

^‡^ Prince Street Medical Practice, Orange, New South Wales, Australia

**Table 1.** Direct acting antiviral (DAA) medication list

| Medicine Active ingredient | Brand name | Genotype |
| --- | --- | --- |
| Daclatasvir | Daklinza | Genotype specific |
| Glecaprevir with pibrentasvir | Maviret | Pan-genotypic |
| Grazoprevir with elbasvir | Zepatier | Genotype specific |
| Ledipasvir with sofosbuvir | Harvoni | Genotype specific |
| Paritaprevir with ritonavir, ombitasvir and dasabuvir | Viekira Pak | Genotype specific |
| Paritaprevir with ritonavir, ombitasvir, dasabuvir, ribavirin | Viekira Pak-RBV | Genotype specific |
| Sofosbuvir | Sovaldi | Genotype specific |
| Sofosbuvir with velpatasvir | Epclusa | Pan-genotypic |

**Table 2.** Clinical definitions used to identify MedicineInsight patients

| Condition | Definition |
| --- | --- |
| HIV infection | Patients were defined as having HIV infection, if they ever had a relevant coded (Docle, Pyefinch) or free text entry in one of the three diagnosis fields. Relevant terms include: HIV, HIV carrier, AIDS, but excludes HIV embryopathy |
| Hepatitis B infection | Patients were defined as having hepatitis B infection, if they ever had a relevant coded (Docle, Pyefinch) or free text entry in one of the three diagnosis fields. Relevant terms include: hepatitis B – immune clearance phase, control phase, escape phase, tolerance phase and hepatitis B carrier |
| Cirrhosis (liver) | Patients were defined as having liver cirrhosis, if they ever had a relevant coded (Docle, Pyefinch) or free text entry in one of the three diagnosis fields. Relevant terms include: hepatic cirrhosis, cirrhosis with acute renal failure, and hepatorenal syndrome, but exclude biliary cirrhosis, cirrhosis – alpha 1 antitrypsin deficiency, haemochromatosis |
| Hepatocellular carcinoma | Patients were defined as having hepatocellular carcinoma, if they ever had a relevant coded (Docle, Pyefinch) or free text entry in one of the three diagnosis fields. Relevant terms include hepatocellular cancer or carcinoma |

**Table 3.** Patients prescribed individual DAA medicines during the study period (1 March 2016–31 August 2018)

| **Medicine active ingredient** | **Patients prescribed a DAA** | |
| --- | --- | --- |
|  | Number | %^†^ |
| Ledipasvir with sofosbuvir | 988 | 43.9 |
| Sofosbuvir | 704 | 31.3 |
| Daclatasvir | 612 | 27.2 |
| Sofosbuvir with velpatasvir | 446 | 19.8 |
| Grazoprevir with elbasvir | 130 | 5.8 |
| Paritaprevir with ritonavir, ombitasvir and dasabuvir | 24 | 1.1 |
| Glecaprevir with pibrentasvir | 19 | 0.8 |
| Paritaprevir with ritonavir, ombitasvir, dasabuvir, ribavirin | 10 | 0.4 |

^†^ The proportion of patients in the DAA study population prescribed the individual DAAs does not add to 100% as patients who were prescribed more than one DAA medicine were counted multiple times.
